# Kindergarten Teachers’ Quality of Work Life in China: A National Empirical Study

**DOI:** 10.3390/ijerph20054596

**Published:** 2023-03-05

**Authors:** Huijuan Di, Huanhuan Li, Yifang Wang

**Affiliations:** 1 Department of Preschool Education, Hebei Normal University, Shijiazhuang 050024, China; 2 College of Educational Science, Xinjiang Normal University, Urumqi 830017, China; 3 Shanghai Institute of Early Childhood Education, Shanghai Normal University, Shanghai 200234, China

**Keywords:** kindergarten teachers, quality of work and life, Chinese teachers

## Abstract

Kindergarten teachers’ quality of work life (QWL) is of great significance in ensuring teacher stability, improvement in education quality, and the development of education. This study used the newly developed and validated tool, the QWL scale for kindergarten teachers (QWLSKT) to investigate QWL among kindergarten teachers in China. The participants comprised 936 kindergarten teachers. Psychometric results showed that the QWLSKT is a reliable and effective scale across six dimensions: health status, interpersonal relationships, working conditions, professional growth, participation in decision making, and leisure life. The Chinese teachers’ evaluation of their professional growth was positive, while their evaluation of their working conditions was negative. Latent profile analysis results showed that a three-profile model had the best fitting model, comprising low, middle, and high profiles in relation to low, medium, or high scale scores, respectively. Finally, the results of hierarchical regression analysis showed that the educational level and establishment of kindergarten teachers, as well as the quality level of kindergartens and their regions, play an important role in the QWL of kindergarten teachers. The results showed that more effective policy and management strategies are needed to improve QWL among kindergarten teachers in China.

## 1. Introduction

The pursuit of high-quality preschool education is globally recognised as worthwhile. In China, teacher-related issues have been a key factor restricting the improvement of preschool education quality and have also been considered a hinderance generally to improving quality overall [1]. In his report on the reform and development of preschool education, Chen Baosheng, the Minister of Education in China, noted that there was a shortage of full-time kindergarten teachers, with 520,000 vacancies [2]. Given the resulting pressure in terms of their work, kindergarten teachers frequently leave their jobs or change jobs. One study found that nearly one third of kindergarten teachers wished to leave their jobs [3], and the turnover rate among kindergarten teachers in one province was as high as 50% [4]. Frequent replacement of teachers is not only detrimental to the establishment of long-term and sustainable attachment between children and teachers, but also directly affects attempts to improve the quality of China’s early childhood education and its sustainable development.

Whether teachers are willing to work in kindergartens is closely related to their salaries and welfare. Research on the job mobility of preschool teachers has found that low wages and low levels of welfare are considered the most common reasons for teachers to leave the field of early childhood education. Working conditions and environment are also important factors affecting teachers’ retention [5,6,7]. Good working conditions can enable teachers to work with ease and satisfaction whereas poor working conditions hinder the retention of competent teachers and the promotion of improvements in teachers’ work efficiency [8]. In recent years, the Chinese government has initiated various safeguarding measures concerning the salaries, welfare provision, and working conditions of kindergarten teachers. For example, the revised draft of the new teachers’ law clearly notes that “the average wage income level of kindergarten teachers should not be lower than or higher than that of local civil servants”. In 2020, China promulgated the Preschool Education Law (Draft for Comment), which legally stipulates that “kindergartens and their organizers should guarantee teachers’ wages and benefits, social insurance benefits, and improve working and living conditions in accordance with relevant state regulations”. However, due to the smaller amount of funding set aside for preschool education in China, the situation confronting kindergarten teachers has not fundamentally changed. It has been noted that low pay, low levels of welfare, and low pay rates form the ‘real crisis’ of preschool education development [9]. Only by addressing these issues is it likely that the quality of teachers can be improved, and sustainable development of preschool education promoted.

Therefore, when considering what is required to promote the professional quality of kindergarten teachers, there needs to be a focus on their living conditions and quality of life to ensure that many more high-quality teachers are retained in early childhood education. Quality of work life (QWL) has been widely concerned scholars in the field of enterprise management since it was first put forward in 1972 by researchers such as Walton (1975), Mirvis and Lawler (1982), etc. It has been considered more important to pay attention to employees’ psychological and material needs to improve work performance than to improve work techniques and methods. Current research on QWL is mainly concentrated in the field of enterprise management, with relevant research in the education industry mainly focused on primary and secondary school teachers. Research on QWL in relation to kindergarten teachers is lacking. This study aimed to address this gap through a nationwide investigation of kindergarten teachers’ QWL using a QWL assessment scale to clarify issues confronting the sustainable development of kindergarten teachers in China and help ensure these issues can be dealt with successfully.

### 1.1. Kindergarten Teachers’ QWL

To understand the current QWL of kindergarten teachers in China, a comprehensive and inclusive indicator system needs to be constructed involving comprehensive and systematic information concerning their current situation. However, QWL is a complex and diversified concept. Different scholars have defined the concept of QWL based on different research fields and theoretical perspectives, with three approaches commonly applied, as follows [10]: The first approach emphasizes personal subjective experience and feelings, such that QWL refers to an individual’s evaluation concerning how much work satisfies his or her needs and makes him or her feel satisfied [11]. The second approach regards QWL as a way to improve the working and living environments; for example, QWL has been defined as a favourable condition and environment in the workplace, which supports and improves employee satisfaction by providing incentives, job security, and development opportunities to employees [12]. The third approach holds that QWL is conceptual and value-based; for example, it has been claimed that QWL is a way of thinking about employees and organizations, with the emphasis on the impact of work on employees and organizational effectiveness and on employees participating in organizational decision-making and problem solving [13]. In considering these approaches and the study context, this study defined QWL in relation to kindergarten teachers as teachers’ comprehensive evaluation and subjective feelings concerning their work [14], which was specifically reflected in teachers’ evaluation of their work (working conditions and professional development), personal situation (health status and leisure life), and organizational engagement (interpersonal relationships; participation in decision-making).

### 1.2. QWL Evaluation of Kindergarten Teachers

As different scholars have different understandings of QWL, there is no consensus on the measurement dimension of QWL. Walton defines QWL according to the eight conditions of employees who make up the ideal QWL: adequate and fair wages, safe and healthy working conditions, opportunity to utilize and develop human abilities, opportunity for career development, social integration of the labour force, constitutionalism of the work organization, quality of work life, and the social relevance of work [15]. The Organisation for Economic Co-operation and Development defined QWL in relation to the following factors: the possibility to work continuously; objective work conditions such as wages, working hours, holidays, actual working conditions, and technology; and subjective working conditions such as job satisfaction, and attitudes towards the organization and supervisors [16]. In a literature review on QWL in relation to kindergarten teachers, specific factors were identified as being most-considered by researchers, namely, teamwork, attitude and cognition, compensation and welfare, social integration, participatory management, organizational commitment, growth and development, job satisfaction, safe and a healthy environment, participatory management, salary, employee participation, welfare opportunities, and rewards [17].

Given this context, it seems that most questionnaires are aimed at all occupations, such as the Leiden Quality of Work Questionnaire [18], with few questionnaires, including the quality of Teacher Work Life Survey, being specifically designed for teachers [19]. The reliability of some current teacher questionnaires is not good. For example, in the teachers’ QWL questionnaire developed by Louis [20], the reliability of one dimension was 0.52. Therefore, this study proposed a new scale to assess kindergarten teachers’ QWL, and then discussed kindergarten teachers’ QWL in China with the aim of promoting even more effective development of early childhood education. In particular, the research questions of this study are as follows:

1. Is the kindergarten teachers’ QWL scale used in this study reliable?
2. How do kindergarten teachers evaluate their QWL?
3. What are the main factors that affect kindergarten teachers’ QWL?

Consequently, this study explores the following assumptions:

**Hypothesis (H1).**  *QWL scale for kindergarten teachers is a reliable scale;*

**Hypothesis (H2).**  *Kindergarten teachers speak highly of their QWL;*

**Hypothesis (H3).**  *Specific factors affect kindergarten teachers’ evaluation of their QWL.*

## 2. Material and Methods

### 2.1. Participants

Kindergarten teachers were recruited from nine provinces in eastern, central, and western China by random sampling, including cities such as Beijing, Shanghai, Tianjin, Zhejiang, Hebei, Anhui, Heilongjiang, Liaoning, and Xinjiang. Among these cities, there are both the most developed cities (such as Beijing) and less developed cities (such as Xinjiang). Finally, 936 valid online questionnaires were returned. According to the administrative division of China, the kindergarten teachers who participated in the survey were distributed in cities (68.1%), counties (13.1%), and countryside (18.9%).

Regarding the types of kindergartens, about 64.6% of the teachers are from public kindergartens run by education departments, 10.6% from public kindergartens run by enterprises and institutions, and 25.8% from private kindergartens. Generally, the Education Department of China divides the quality of kindergartens into different grades (demonstration kindergartens, first-class kindergartens, secondary kindergartens, grade III kindergartens, or unrated kindergartens). Further, data concerning teachers’ age, teaching years, professional titles, education level, establishment (long-term employment as opposed to temporary employment or ‘non-establishment’), and geographical area were also collected (see Table 1).

### 2.2. Measures

#### 2.2.1. Development of the QWL Scale for Kindergarten Teachers (QWLSKT)

The QWLSKT adopts three frameworks (personal level, work level, and organization level) and six dimensions (health status, interpersonal relationships, working conditions, leisure life, professional growth, and participation in decision-making). The formulation of the theoretical framework of the QWLSKT has a solid foundation.

First of all, literature review. We comprehensively consulted the domestic and foreign policy documents and related studies on the QWL, and learned the concept definition and evaluation dimension of the QWL. Then, the dimensions of QWL put forward by domestic and foreign scholars are sorted out and the frequency of these dimensions is counted.

Second, theoretical construction. As can be seen from the above, different scholars have different entry points for QWL, and the dimensions of the QWL are also different. Cummins and Huss (1985) pointed out that the QWL can be defined and conforms to the actual situation as long as it is considered from three aspects: individual, work and organization [21]. Therefore, this study adopts the viewpoint of Cummins and Huss, and divides the QWL into individual level, work level, and organization level. The dimension with higher frequency among the dimensions of QWL combed in the early stage is taken as the initial dimension of the scale. The QWL scale with three structures, nine dimensions and 38 indicators was initially developed. Including the personal level: respect and care, work-life balance, career growth and development; benefits, working environment and working conditions at the working level; Interpersonal relations, leadership management and decision-making participation at the organizational level.

Third, expert consultation. We invited 15 experts in the field of preschool education, and finally agreed to participate in this study. There are 12 experts (2 university professors, two kindergarten directors, four teachers with more than 15 years’ work experience and four teachers with more than 10 years’ work experience). In this study, an expert questionnaire on the QWL of kindergarten teachers was used to ask for experts’ opinions on the initially constructed index system. The questionnaire is mainly composed of three parts. The first part is the personal information of experts. The second part is the evaluation of the importance of the index system by experts. Experts need to fill in their own reasonable grade scores after each index, point out the indexes that are suggested to be modified, added or deleted, and explain the specific reasons. The third part is two open-ended questions, which are: “What elements do you think should be included in the QWL of kindergarten teachers?”, “your other opinions and suggestions on the indicator system.” After two rounds of expert consultation, experts agree with the index system and put forward reasonable suggestions: some dimensions were deleted, such as “respect and care”, which were just a small aspect of QWL, and it is not enough as a dimension; some dimensions have been added, such as “Health status”, because experts believe that the physical and mental health of kindergarten teachers is an important factor in QWL. Some dimensions have been revised, for example, the expression of “work-life balance” is more general, and the content points to the personal space and leisure life of teachers. Now it has been revised as “leisure life”. Some dimensions have been merged, such as “working environment” and “working conditions”, which overlap in content and merge into “working conditions”. The Scale was revised by experts, with three constructs and six dimensions, and 29 indicators.

Fourth, focus group interviews. The researcher invited four graduate students and two undergraduate students majoring in preschool education to review and revise the content and language expression of the project, so as to evaluate the content validity of the questionnaire. Try to use accurate words, concise sentence and easy to understand for each survey item, so as to reduce the pressure of filling in and answering for kindergarten teachers. At the same time, try to make language expression neutral and objective, and avoid the emergence of social identity effect.

Fifth, pilot research. According to the principle of convenient sampling, 120 kindergarten teachers were recruited to test the scale. Teachers all think that the test questions are consistent with the work practice of kindergarten teachers and the expression habits of kindergarten teachers. According to the preliminary analysis of the test data, five questions were deleted because they did not conform to the principles of item analysis and factor analysis. The final scale of kindergarten teachers’ QWL includes three structures, six dimensions and 24 indicators (see Appendix A).

#### 2.2.2. Data Obtained Using a Demographic Questionnaire and the QWLSKT

The kindergarten teachers who participated in this study completed two kinds of questionnaires. One is the questionnaire of demographic information of teachers, including teachers’ age, teaching years, professional titles, education level, and establishment, as well as the nature, geographical area, and the level of the kindergarten where the teachers are located. The other is the QWLSKT questionnaire, which includes a total of 24 test items, using a five-point Likert scale (1 = Strongly disagree; 5 = Strongly agree). QWLSKT’s projects reflect all aspects of teachers’ QWL. (for example, “I am very satisfied with the office conditions of kindergartens” and “I have the right to speak and decide on kindergarten management related to my major”).Use the statistical average of all the corresponding items contained in each dimension to calculate the scores of the six dimensions. Higher average score of a teacher means better QWL of a kindergarten teacher.

### 2.3. Procedures

The whole research process was carried out in strict accordance with ethical procedures. First, the research was approved by the ethics review committee of the university where the author works, and the ethics approval number is HR 072-2020. Second, all kindergarten teachers and principals involved in the survey expressed their informed consent. Third, at the beginning of the questionnaire, participants were provided a brief introduction to the contents of the survey: The research was anonymous, and the results of the survey were for research purposes only. Teachers participating in the survey could withdraw from the research at any time without having to bear the consequences.

The questionnaire was distributed through the online questionnaire survey platform www.wjx.cn, accessed on 15 September 2022. A total of 964 kindergarten teachers completed the questionnaire. A completed questionnaire was excluded because of the following reasons: (1) teachers completed the questionnaire in a short time, that is, less than 200 s, (2) the demographic information was complete, or (3) more than 90% of the answers were the same. The final data set contained 936 valid questionnaires, and the recovery rate was 97%.

### 2.4. Data Analysis

The data were analysed by Amos 21.0, SPSS 26.0 (IBM, Shanghai, China) and Mplus 7.4 software. First, the psychometric characteristics of the QWLSKT were analysed by item analysis, exploratory factor analysis (EFA) and confirmatory factor analysis (CFA) to explore and verify the construct effectiveness and reliability of the QWLSKT. Second, a latent profile analysis was used to generate a latent profile of Chinese kindergarten teachers’ QWL. Third, teachers’ main prediction of their QWL were checked by hierarchical regression analysis. Finally, with the QWLSKT grades as the independent variable, we explored the differences between the six dimensions and the total scores of the QWLSKT in relation to the teachers’ QWL using ANOVA analysis.

## 3. Results

### 3.1. Psychometric Characteristics of the QWLSKT

#### 3.1.1. Item Analysis

Item analysis was used to test the quality of scale items. After analyzing these items with factor load and total correlation method, it was decided to keep all these items for the following reasons. First of all, the factor loads of all projects are between 0.53 and 0.85, both of which were greater than 0.45. Second, the correlation coefficient between each item and the total score was between 0.52 and 0.75, which was greater than 0.40. Third, the internal consistency coefficient of the questionnaire was 0.944, and the reliability coefficient of the questionnaire after all the items had been deleted was not higher than 0.944, indicating that the dimensions and attributes to be measured for each item had high homogeneity. Finally, the total scores of the items were ranked from low to high and from high to low, with 27% selected as the cut-off point for the low and high groups, respectively. This was determined following mean difference test results that showed that the CR value of each item was between 17.14 and 30.41 (*p* < 0.001), which was much higher than 3.00, indicating that the score of the high group was significantly higher than that of that low group, and that the item discrimination was higher.

#### 3.1.2. EFA

SPSS 26.0 software was used to decompose the first half of the sample (*n* = 468) by principal axis. First the data were very suitable for factor analysis, Kaiser-Meyer-Olkin = 0.838 and Bartlett’s test of sphericity χ2 = 5024.991 (df = 276, *p* < 0.001). Second, using principal axis factorization and the direct oblimin method, the six-factor model for this scale was generated. It explained 67.46% of the total variance, which demonstrated the structural validity of the QWLSKT was satisfactory. The characteristic value of the six factors were 5.84, 4.06, 2.20, 1.59, 1.36, and 1.15, respectively. Third, the factor loadings of the items were from 0.55 to 0.86, which were more than 0.45; the common degrees were between 0.50 and 0.83, which were more than 0.20 (see Table 2). Moreover, the common factors within the factor structure were also relatively stable, so all items were reserved.

Further, the results of exploratory factor analysis showed that the variance interpretation rate of the first factor was 5.84%, including four items, which mainly referred to the situation where the teachers participated in professional training, academic groups, and scientific research activities, so it was termed ‘professional growth’ (Table 2). The explanation rate of variance of the second factor was 4.06%, including five items, which mainly referred to the right of the teachers to participate in the teaching and management of the kindergarten, and it was termed ‘participation in decision-making’. The variance interpretation rate of the third factor was 2.20%, including five items, which mainly referred to the interpersonal relationships between the teachers and children’s parents, colleagues, principals, and other roles, so it was termed ‘interpersonal relationships’. The explanation rate of variance of the fourth factor was 1.59%, including four items, which mainly referred to the teachers’ salaries and office conditions, so it was termed ‘working conditions’. The explanation rate of variance of the fifth factor was 1.36%, including three items, which mainly referred to the time, type, and quality of the teachers’ leisure and entertainment, so it was termed ‘leisure life’. The explanation rate of variance of the sixth factor was 1.15%, which mainly referred to the teachers’ physical, mental, and sleep conditions, so it was termed ‘health status’. The above six factors were consistent with the hypothetical dimensions. Therefore, the QWLSKT was retained with the dimensions of health status, interpersonal relationships, working conditions, leisure life, professional growth, and participation in decision-making.

#### 3.1.3. CFA

**Figure 1:**
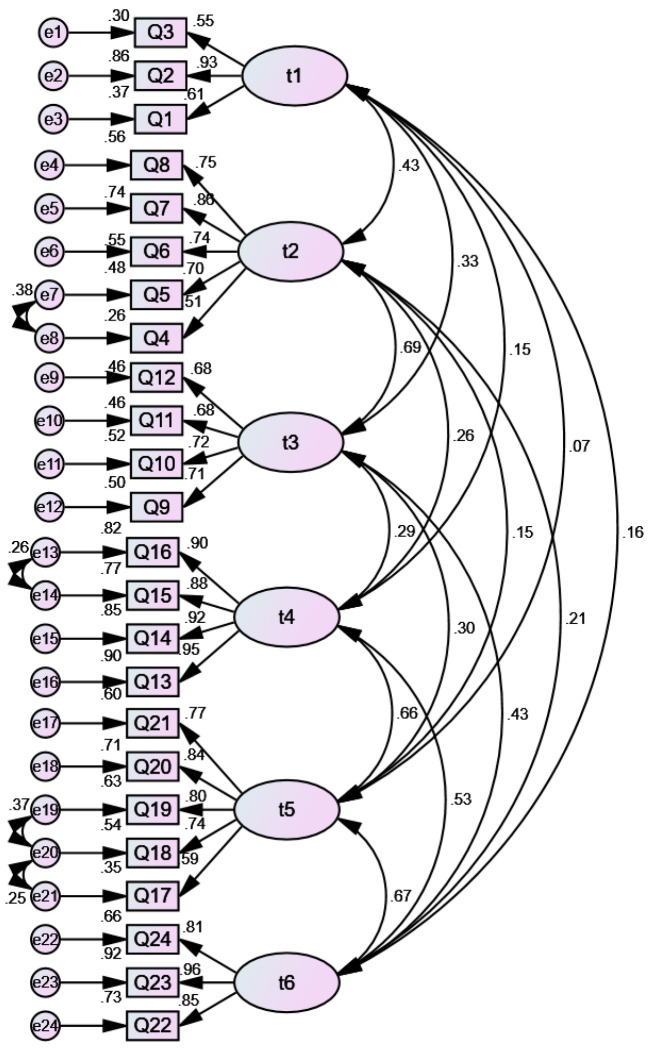
CFA of the QWLSKT. Model fit: χ2 = 691.447, *df* = 235, *p* < 0.000, CFI = 0.91, TLI = 0.90, IFI = 0.91, RMSEA = 0.067 (90% CI [0.61, 0.73]), AIC = 821.447, BIC = 1086.494. CFA, confirmatory factor analysis; QWLSKT, quality of work life scale for kindergarten teachers; CFI, confirmatory factor index; TLI, Tucker-Lewis index; IFI, incremental fit index; RMSEA, root mean square error of approximation; AIC, Akaike information criteria; BIC, Bayesian information criteria.

The second part of the data (*n* = 468) is analysed by CFA to confirm the six-factor structure of the EFA results put forward before. In accordance with recognized standards, when χ2/df is smaller than 5, the model fitting effect is acceptable. The Tucker-Lewis index (TLI) comparative fit index (CFI), and incremental fit index (IFI) indicator values can be between 0 and 1. The closer the value is to 1, the more appropriate the model is. Usually, when these indexes are greater than 0.9, the model fitting effect is good. The closer the suggested critical value for the root mean square error of approximation (RMSEA) is to 0, the more fitting the model is, as a value less than or equal to 0.08 is usually considered the standard [22]. The first-order CFA results confirmed that the six-factor structural model is in good agreement with the data. The model showed high fitting degree, which indicates that the model fits well, χ2 = 691.447, df = 235, *p* < 0.000, CFI = 0.91, TLI = 0.90, IFI = 0.91, RMSEA = 0.067 (90% confidence interval [CI] [0.61, 0.73]), Akaike information criteria (AIC) = 821.447, Bayesian information criteria (BIC) = 1086.494 (see Figure 1).

#### 3.1.4. Reliability Analysis

Table 3 indicated that, the Cronbach α value of QWLSKT was 0.95, and the reliability of all factor was above 0.82, which indicates that the reliability is very good. The range of split-half reliability is 0.70 to 0.91, which indicates that the internal consistency reliability of the scale is good [23].

### 3.2. Descriptive Analysis of the QWLSKT

Overall, kindergarten teachers in China evaluated their QWL positively, and the average total score of the QWLSKT was higher than 3.5 (M = 3.61, SD = 0.74), but their scores in certain dimensions were mixed. Specifically, professional growth had the highest score (M = 4.00, SD = 0.81), followed by interpersonal support (M = 3.82, SD = 0.80), health status (M =3.82, SD = 0.88), and participation in decision-making (M = 3.53, SD = 0.91), while the leisure life (M = 3.22, SD = 1.07) score was lower than the overall average score and working conditions had the lowest score (M = 3.18, SD = 0.92). Among the 24 test items, the one with the lowest scores included: “I am very satisfied with my salary and welfare” (M = 2.72, SD = 1.15); “I have enough leisure time” (M = 3.09, SD = 1.18); “Teachers’ leisure activities are rich and diverse” (M = 3.13, SD = 1.20); and “I am very satisfied with my housing conditions” (M = 3.20, SD = 1.18), which indicated that the teachers had negative views on these four items.

### 3.3. Latent Profile Analysis of the QWLSKT

**Figure 2:**
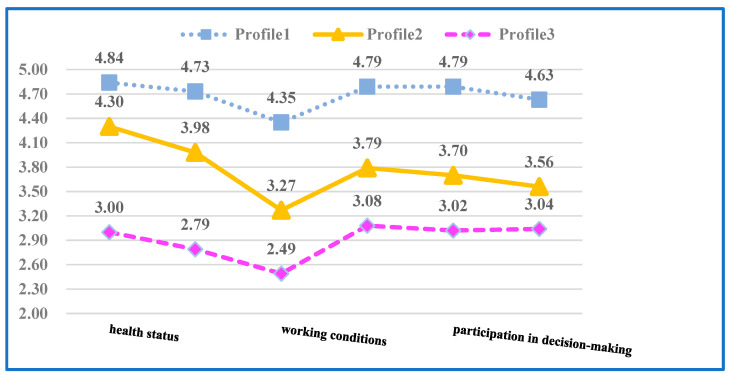
The three profiles based on mean evaluation scores on the quality of work life scale for kindergarten teachers (QWLSKT) (N = 936). Profile 1 = high level (*n* = 383); profile 2 = middle level (*n* = 384); profile 3 = low level (*n* = 309). AIC, Akaike information criteria; BIC; Bayesian information criteria; LRT, likelihood-ratio test; BLRT, bootstrap likelihood ratio test.

In order to explore the potential characteristics of kindergarten teachers’ QWL, various models with different numbers of ‘latent’ categories were estimated. Based on the results of fitting indicators of each model, the three-profile model was considered to be the best model. Table 4 indicates the three-profile model, which was chosen because it had statistically significant likelihood-ratio test result values (*p* < 0.05) when compared with the five-profile model. Compared with the two-profile model, it had lower AIC (13,284.733), BIC (13,444.506), and aBIC (13,339.701) values, and the entropy value was greater than 0.8. Considering the best latent profile analysis practices related to the interpretability and simplicity of the identified configuration files, the three-profile model was superior to the four-profile model. The apparent validity of the profile was also evaluated subjectively, with the three-profile structure, as noted being found the most appropriate. Among the six dimensions of QWLSKT, the proportion of the three determined contours and their response probability are shown in Figure 2. Below, we provide a brief overview of these three profiles, representing 40.1%, 26.1%, and 33.0% of the participants, respectively.

The three contours have different characteristics in six dimensions (see Table 4 and Figure 2). Teachers with Profile 1 scored the highest in six dimensions; this number of teachers were categorised as a ‘high level’ group, with 309 (33.0%) teachers comprising this group. The second category of teachers scored relatively lower on the six dimensions than those of the first type of teachers; these teachers were categorised as a ‘middle level’ group, with 383 (40.1%) teachers comprising this group. Finally, teachers in Profile 3 scored the lowest on the six dimensions; this team was categorised as a ‘low level’ group, with 244 (26.1%) teachers comprising this group.

Chi-square analysis was used to test the differences among demographic variables of the low, middle, and high levels. Chi-square tests in Table 5 show that these three situations were distinguished according to years of teaching experience. (χ2 (10, N = 936) = 19.655, *p* < 0.05, Cramer’s V = 0.102 and Somer’s d = −0.011), professional title (χ2 (10, N = 936) = 25.176, *p* < 0.01, Cramer’s V = 0.116 and Somer’s d = −0.044), education (χ2 (8, N = 936) = 35.126, *p* < 0.000, Cramer’s V = 0.137 and Somer’s d = −0.041), establishment (χ2 (4, N = 936) = 11.147, *p* < 0.05, Cramer’s V = 0.077 and Somer’s d = 0.051), kindergarten quality level (χ2 (8, N = 936) = 25.540, *p* < 0.01, Cramer’s V = 0.114 and Somer’s d = −0.088), and geographical area (χ2 (6, N = 936) = 17.940, *p* < 0.01, Cramer’s V = 0.098 and Somer’s d = −0.110).

Profile 1: low level. Main features in this group include: years of teaching experience less than 5 (12.4%), no professional title (13.2%), education level to senior high or below (0.8%), non-establishment (13.6%), kindergartens at grade III or unrated (4.6%), and located in the countryside (3.7%). These results showed that teachers who had short teaching experience, who were non-establishment teachers, with a lower level professional title and educational background, in Grade III or unrated kindergartens, and in the countryside were more likely to make negative comments about their QWL.

Profile 2: middle level. Features mainly in this group include: teaching for 6–15 years (11.7%), intermediate professional title (9.5%), education level to junior college (18.8%), establishment (15.9%), secondary kindergartens (3.2%), and located in the county (6%). These results showed that teachers with 6–15 years of teaching experience, an intermediate professional title, and a college degree, who were establishment teachers, in secondary kindergartens, and in the county were more likely to have a middle level comment on their QWL.

Profile 3: high level. Main features in this group include: more than 16 years of teaching experience (5.1%), senior professional title (2.4%), education level to bachelor’s degree or above (14.2%), establishment (10.9%), demonstration or first-class kindergartens (24.7%), and located in the city (24.4%). These results showed that teachers who teach for a long time, with a senior professional title, and with a bachelor’s degree or above, who are establishment teachers, in demonstration or first-class kindergartens, and in the city were more likely to have a positive comment on their QWL.

### 3.4. Predictors of the QWLSKT Evaluation by Teachers

The variables related to teachers’ evaluation of QWL were discussed through the Spearman correlation analysis. As shown in Table 6, the following variables were significantly related to the QWLSKT total score (*p* < 0.01). Title (no title, junior, intermediate, and senior), education level (senior high or below, junior college, and bachelor’s degree or above), geographical area (city, county, and countryside), and kindergarten quality grade (demonstration kindergarten, first-class kindergarten, secondary kindergarten, and grade III or unrated kindergarten).

Lastly, a 3-step hierarchical regression analysis was conducted using the enter method to predict the total score of the QWLSKT. The independent variables were divided into three levels: (1) personal variables: education and establishment; (2) kindergarten variables: quality level of kindergarten; and (3) regional factors: geographical area where the kindergarten is located. Then the teachers’ personal variables were entered in step 1, the kindergarten variables in step 2, and the regional variables in step 3.

The changes in R2 between the three steps showed that: (1) the teachers’ educational level and establishment can jointly explain 1.6% of the total QWLSKT score; (2) the kindergarten quality grade factor can significantly predict the total score of QWLSKT 2.3%; and (3) geographical location can explain 0.9% of the total score change in QWLSKT. The quality level of the kindergartens and regional factors were negative predictors of teachers’ QWL.

In other words, the hierarchical regression analysis showed that the quality level of kindergarten was a significant predictor for China’s teachers to evaluate their QWL. Specifically, the QWL of high-quality kindergarten teachers was higher than that of low-quality kindergarten teachers. Teachers’ personal factors, such as education and establishment, had a significant negative impact on teachers’ evaluation of their QWL, accounting for 1.6% of the total change. Geographical factors also affected teachers’ QWL, and teachers in cities scored higher than those in rural areas (Table 7).

## 4. Discussion

This research mainly aimed at developing and validating the QWLSKT by evaluating kindergarten teachers’ QWL in China. The results showed that this scale has good psychometric characteristics and provides an effective measurement tool for QWL of kindergarten teachers, supporting H1. In order to develop and determine the scale items, teachers’ opinions on QWL were interviewed. Moreover, this investigation and study provided an empirical basis for assessing teachers’ QWL, and the results were positive in terms of their health status, interpersonal support, professional growth, and participation in decision-making, thus supporting H2, while teachers’ working conditions and leisure life need to be improved. Finally, the grade of kindergarten quality was found to be an important predictive index of teachers’ QWL, supporting H3.

### 4.1. The QWLSKT Was Reliable and Valid

To determine kindergarten teachers’ QWL, this research developed and verified the QWLSKT, which has six dimensions and showed satisfactory structural validity, for the case of China. The development of this QWLSKT not only drew extensively on relevant research on the evaluation of teachers’ and kindergarten teachers’ QWL both in China and in other countries, but it also noted the professional characteristics of kindergarten teachers in China. It was validated after being revised and verified by local authoritative experts and has high content validity. Meanwhile, EFA and CFA results showed that the items included in the six dimensions were consistent with the original concept of the scale design, and that the fitting index of the structural equation model was good, indicating that the scale had good structural validity. This research also found QWLSKT had desirable reliability and internal consistency. Therefore, in terms of its psychometric properties, the QWLSKT was found to be a suitable tool to evaluate teachers’ QWL in China. Specifically, it provides a comprehensive framework reflecting the QWL of kindergarten teachers as well as key evidence on core aspects of teachers’ QWL, which enables researchers and policymakers to engage more effectively with issues related to the QWL of kindergarten teachers.

### 4.2. Kindergarten Teachers’ QWL

This research found that most teachers (57.8%) rated their QWL positively, indicating that teachers’ QWL is at an acceptable level in China. Specifically, teachers spoke highly of their professional development. This indicates that kindergarten teachers pay attention to their own growth and development, this is possibly associated with China’s continuous emphasis on improving the quality of childhood education for the past few years [24]. Since 2010, China has implemented a national training program for kindergarten teachers, which aims to provide targeted training for kindergarten teachers to improve the teaching ability and professional level of all kindergarten teachers [25]. Coincidentally, in a study conducted in India, most teachers were satisfied with the opportunity to develop human capabilities in institutions [26].

Further research found that kindergarten teachers provided the lowest evaluation for their working conditions. Moreover, in the dimension of teachers’ working conditions, the lowest score is “I am very satisfied with my salary and benefits” (m = 2.72, SD = 1.15). Hamidi and Mohamadi (2012) also found that teachers’ opinion on fair and adequate payment is lower, and their salary is not satisfactory and not associated with their job [27]. The working conditions of kindergarten teachers have an important impact on their physical and mental status, teaching quality, and turnover intention [28], and are the focus of policy reform and practical research to improve the quality of preschool education around the world. Before the development of QWLSKT, this study interviewed several teachers. Teachers generally expressed their desire to improve their rights, interests, working conditions, and social status. However, due to historical, conceptual, and other subjective reasons, some local governments and relevant departments in China have long lacked scientific understanding of the nature, orientation, and important role of preschool education. As a result, the status and professionalism of kindergarten teachers are not recognized, which directly affects their social reputation, welfare, and QWL.

### 4.3. Factors Influencing Teachers’ Evaluations of Their QWL

This study identified three profiles concerning teachers’ QWL (low, middle, and high), it reflects the demographic characteristics of teachers in China. Teachers with short teaching experience, who were non-establishment, who had lower levels of professional title and educational background, who worked in Grade III or unrated kindergartens, and who worked in the countryside were more likely to evaluate their QWL negatively. On the other hand, teachers who had taught for a long time, with a senior professional title, with a bachelor’s degree or above, who were establishment teachers, who worked in demonstration or first-class kindergartens, and who worked in the city were able to evaluate their QWL positively.

Generally speaking, teachers with long teaching experience are relatively richer in teaching experience. Furthermore, long teaching experience not only involves having taught for longer but also is likely to reflect a higher professional reputation and social status, with such teachers having a stronger voice and greater decision-making power in the affairs of the kindergarten. Research showed that there is a significant correlation between the length of service of the respondents and their perception levels of overall QWL in the teaching environment [29]. Compared with rural areas, urban areas have advantages in terms of economic development and investment in education funds. Such areas attract better quality teachers as well as better guarantees concerning the salaries and welfare of teachers to a certain extent, so urban teachers’ QWL tends to be relatively high. A study on rural teachers in QWL found that the quality of teachers’ work and life was affected by the remoteness or underdevelopment of their workplace, and rural teachers were affected in accommodation, water supply, material condition of the school, teaching and learning materials, distances from towns, allowances, and promotions [30]. Moreover, the research found that public school teachers with establishment rated their QWL higher. Public kindergartens in China are state-owned assets, which aim at ensuring greater welfare and better quality [31]. Therefore, they employ more establishment teachers, whereas most teachers in private kindergartens are non-establishment teachers. It is difficult for private kindergartens to ensure the rights and interests of teachers are protected in terms of salary level, welfare benefits, professional title evaluation, and recruitment, training, and further education. Therefore, establishment teachers are typically found in good-quality public kindergartens.

### 4.4. Disparities Revealed through the QAWLSKT

This study found that the educational level and establishment of kindergarten teachers, as well as the quality level of kindergartens and their regions, play an important role in the QWL of kindergarten teachers. Previous research found that the higher the education level of teachers, the better their quality of life [32]. Kindergartens are crucial to preschool education and reflect developments in preschool education to a certain extent. Kindergarten teachers are likely to evaluate their QWL according to their views on the kindergarten where they work. Furthermore, the higher the quality level, the better the financial and resource support provided by the government [33]. For example, the Beijing Kindergarten Quality Supervision and Evaluation Standards (Trial) clearly states that ”the supervision and evaluation results will be publicized and will serve as an important basis for kindergarten performance management, per student subsidies, commendation and rewards, and cadre assessment” [34]. Therefore, the government is more inclined to provide financial support to high-level kindergartens, which brings about the Matthew effect; that is, the better the kindergartens with better quality, the worse the kindergartens with worse quality [35]. A recent study by Dorji and Patcharin (2019) found that teachers in remote and difficult schools were emotionally affected due to separation from their family and were dissatisfied about the lack of modern urban facilities [36]. This is probably the reason why teachers in low-quality and rural areas kindergartens have a low opinion of their QWL.

## 5. Conclusions, Limitations, and Implications

The purpose of this study is to use QWLSKT to evaluate the QWL of kindergarten teachers in China to help kindergarten teachers understand their QWL. First, the QWLSKT in terms of its six dimensions, namely, health status, interpersonal relationships, working conditions, professional growth, participation in decision-making, and leisure life, was found to be reliable and valid. The teachers rated professional growth the highest, followed by health status, interpersonal relationships, participation in decision-making, leisure life, and working conditions. Second, we determined three teacher evaluation profiles that reflected the three levels of teachers’ QWL. Teachers’ background and region had a remarkable influence on their QWL evaluation and reflected various social factors that affected their QWL. Specifically, teachers who had taught for a long time, with a senior professional title, with a bachelor’s degree or above, who were establishment teachers, who worked in demonstration or first-class kindergartens, and who worked in the city were able to evaluate their QWL positively. Finally, the results of regression analysis show that the educational level and establishment of kindergarten teachers, as well as the quality level of kindergartens and their regions, play an important role in the QWL of kindergarten teachers, and it is also a key issue in terms of restricting their QWL.

There were some limitations in this research. First, this study only assessed kindergarten teachers’ QWL from the teachers’ perspective. Future research should include more stakeholders and comprehensively examine the present situation of the QWL of kindergarten teachers. Second, this study only collected the QWLSKT from the teachers’ subjective point of view, which may produce a social approval effect. Thus, the educational panel data should be added as a supplement in future research. Third, the research sampling was unbalanced. For example, the majority of teachers came from cities (68.1%), with very few coming from the counties and the countryside. This provides a direction for further research. Fourth, this research only took the method of a questionnaire survey to examine kindergarten teachers’ QWL. Self-assessment questionnaires are limited in that it is difficult to avoid the impact of the social approval effect on participants as they complete the questionnaires. In future research, the additional use of situational tests, classroom observation, data access, and other methods is recommended to help fully understand kindergarten teachers’ QWL.

This study is the first to conduct a nationwide survey for the development and verification of the scale to evaluate kindergarten teachers’ QWL from the teachers’ perspective. This new QWLSKT can be used as an effective research tool to have a better understanding of the quality of kindergarten teachers’ QWL. It provides evidence support to help address areas of concern and supports targeted improvement in kindergarten teachers’ QWL in terms of their health status, interpersonal relationships, working conditions, professional growth, participation in decision-making, and leisure life. Researchers and policymakers can systematically engage with an informed understanding of kindergarten teachers’ QWL life through the framework of the QWLSKT, which has positive implications for improving kindergarten teachers’ QWL in China and for more appropriate policy formulation in other regions. The study findings can also enable kindergarten teachers to become knowledgeable concerning their QWL, which may stimulate their professional development motivation and ultimately further improve their professional level. However, some ongoing issues were identified: working conditions and the leisure life of kindergarten teachers were not highly rated, which indicates that relevant and targeted policies are needed to support improvement in kindergarten teachers’ QWL in these areas. Regarding regional differences, China is a big country, with significant differences in terms of economic development among different areas. Teachers in highly developed regions and kindergartens with higher quality are more likely to enjoy rich resources. Therefore, underdeveloped areas need to pay special attention to policies. To achieve higher quality in kindergarten teachers’ QWL, relevant early childhood education-related policies need to be adopted and implemented.

## Figures and Tables

**Table 1:** Participants’ characteristics (N = 936).

Demographic Characteristics	N	N%
Age		
≤20	18	1.9%
21–25	347	37.1%
26–30	204	21.8%
31–35	164	17.5%
36–40	94	10.0%
≥40	109	11.6%
Years of teaching experience		
≤5	515	55.0%
6–15	270	28.8%
≥16	151	16.1%
Professional title		
No title	572	61.1%
Primary	168	17.9%
Middle	115	12.3%
Senior	81	8.7%
Education		
Senior high or below	67	7.0%
Junior college	397	42.4%
Bachelor’s degree or above	473	50.5%
Establishment		
Yes	360	38.5%
No	576	61.5%
Geographical area		
City	636	67.9%
County	122	13.0%
Countryside	178	19.0%
Kindergarten type		
Public kindergartens run by the education department	594	63.5%
Public kindergartens run by enterprises and institutions	100	10.7%
Private kindergartens	242	25.9%
Kindergarten quality grade		
Demonstration kindergarten	454	48.5%
First-class kindergarten	193	20.6%
Secondary Kindergarten	79	8.4%
Grade III or unrated kindergarten	210	22.4%

**Table 2:** Exploratory factor analysis results for the QWLSKT.

Item	Factor Loading						Extraction
	1 (4)	2 (5)	3 (5)	4 (4)	5 (3)	6 (3)	
16	0.86						0.78
13	0.85						0.75
14	0.83						0.75
15	0.82						0.72
19		0.84					0.75
18		0.83					0.73
20		0.77					0.66
21		0.64					0.56
17		0.57					0.50
5			0.85				0.75
7			0.76				0.72
4			0.74				0.58
6			0.69				0.60
8			0.55				0.59
9				0.79			0.68
10				0.74			0.60
11				0.73			0.61
12				0.60			0.55
23					0.85		0.83
22					0.83		0.77
24					0.80		0.72
2						0.85	0.78
1						0.81	0.67
3						0.64	0.54

Note: the extraction method was principal axis factoring with a direct oblimin rotation; rotation converged after 9 iterations.

**Table 3:** Item means, standard deviations, and reliability of the quality of work life scale for kindergarten teachers (QWLSKT).

Dimension	M	SD	Cronbach’s Alpha (α)	Split-Half Reliability
Total score of the QWLSKT	3.61	0.69	0.95	0.91
Health status	3.82	0.88	0.82	0.70
Interpersonal relationships	3.82	0.80	0.90	0.84
Working conditions	3.18	0.92	0.83	0.81
Professional growth	4.00	0.81	0.92	0.91
Participation in decision-making	3.53	0.91	0.87	0.82
Leisure life	3.22	1.07	0.91	0.76

**Table 4:** Latent profile analysis model-fit statistics of the potential models.

Model	AIC	BIC	aBIC	LRT	BLRT (*p*)	Entropy	Class Probability
2	14,097.578	14,189.568	14,129.226	0.000	0.000	0.831	0.484/0.516
3	13,650.572	13,776.454	13,693.880	0.036	0.000	0.814	0.401/0.261/0.330
4	13,284.733	13,444.506	13,339.701	0.000	0.000	0.868	0.233/0.115/0.329/0.323
5	13,146.751	13,340.416	13,213.379	0.689	0.000	0.910	0.230/0.102/0.150/0.284/0.234

**Table 5:** Demographic characteristics in terms of the three profiles.

Variable	Profile 1 *n* = 309	Profile 2 *n* = 383	Profile 3 *n* = 244	χ2	Cramer’s V	Somer’s d
Years of teaching experience				19.655 *	0.102 *	−0.011 *
≤5	12.4%	22.9%	19.7%			
6–15	9.0%	11.7%	8.3%			
≥16	4.7%	6.3%	5.2%			
Professional title				25.176 **	0.116 **	−0.044 **
Junior	15.3%	27.6%	24.3%			
Middle	8.2%	9.5%	6.2%			
Senior	2.4%	3.7%	2.4%			
Education				35.126 ***	0.137 ***	−0.041 ***
Senior high or below	0.8%	2.6%	7.0%			
Junior college	8.5%	18.8%	42.4%			
Bachelor’s degree or above	3.7%	15.1%	14.2%			
Establishment				11.147 *	0.077 *	0.051 *
Yes	11.6%	15.9%	10.9%			
No	13.6%	23.3%	19.8%			
Geographical area				17.940 **	0.098 **	−0.095 **
City	17.9%	25.6%	24.3%			
County	3.8%	6.0%	3.2%			
Countryside	4.3%	9.3%	5.5%			
Kindergarten quality grade				25.540 **	0.114 **	−0.088 **
Demonstration kindergarten	11.9%	18.4%	18.3%			
First-class kindergarten	6.2%	8.0%	6.4%			
Secondary Kindergarten	3.4%	3.2%	1.8%			
Grade III or unrated kindergarten	4.6%	11.3%	6.5%			

Note: profile 1: low level; profile 2: middle level; profile 3: high level. * *p* < 0.05, ** *p* < 0.01, *** *p* < 0.001.

**Table 6:** Correlation analysis between contributors and the quality of work life scale for kindergarten teachers (QWLSKT).

Variables	1	2	3	4	5	6	7	8	9
1. Total score of the QWLSKT	——								
2. Age	−0.020	−0.053	——						
3. Years of teaching experience	−0.026	−0.087 **	0.832 **	——					
4. Professional title	−0.062	−0.030	0.522 **	0.534 **	——				
5. Education	−0.115 **	−0.044	0.291 **	0.286 **	0.375 **	——			
6. Establishment	0.080 *	−0.024	−0.357 **	−0.358 **	−0.598 **	−0.330 **	——		
7. Geographical area	−0.080 *	0.150 **	−0.010	−0.094	−0.005	−0.089 **	−0.160 **	——	
8. Kindergarten type	−0.020	0.014	−0.226 **	−0.181 **	−0.302 **	−0.255 **	0.443 **	−0.065 *	——
9. Kindergarten quality grade	−0.117 **	0.086 **	−0.200 **	−0.221 **	−0.188 **	−0.234 **	0.123 **	0.403 **	0.267 **

Note: * *p* < 0.05, ** *p* < 0.01.

**Table 7:** Summary of hierarchical regression analyses predicting teacher evaluation.

	*β*	*R^2^*	Δ*R*^2^	F Value
Step 1		0.016	0.016	7.700 ***
Education	−0.035 ***			
Establishment	−0.113			
Step 2		0.039	0.023	12.748 ***
Education	−0.044			
Establishment	−0.152 ***			
Kindergarten quality grade	−0.158 ***			
Step 3		0.049	0.009	7.952 ***
Education	−0.038			
Establishment	−0.157 ***			
Kindergarten quality grade	0.147 ***			
Geographical area	0.040			

Note: *** *p* < 0.001.

## Data Availability

The data provided in this study can be requested by contacting the author. These data are confidential for ethical reasons.

## Appendix A

**Table A1:** Quality of work life scale for kindergarten teachers.

Dimension	Definition	Indicators
A Health status	Teachers’ objective situation and subjective experience in physical health	Good health; be full of energy; sleep well
B Interpersonal relationships	Types of communication and relationships related to teachers’ work and life	relationships with family; relationships with friends; relationships with parents of children; relationships with colleagues; relationships with the director;
C Working conditions	Resources, environment, and conditions for teachers to live a normal life and complete teaching work	Wage income; housing conditions; office conditions; working hours
D Professional growth	Professional growth and self-development of teachers in teaching	Professional training; career planning; self-reflection; education and scientific research;
E Participation in decision-making	Professional autonomy and decision-making rights enjoyed by teachers in their work	teaching autonomy; right to professional growth; right to park management; right to leisure and entertainment
F Leisure life	Leisure and entertainment life of teachers in their spare time	Leisure time; leisure type; leisure quality
